# Systemic lupus erythematosus complicated by thrombotic microangiopathy with atypical HUS features: A case report

**DOI:** 10.1097/MD.0000000000046412

**Published:** 2026-07-17

**Authors:** Yuting Lin, Jinjian Guo

**Affiliations:** a Department of Cardiology, The Second People’s Hospital Affiliated to Fujian University of Traditional Chinese Medicine, Fuzhou, Fujian, China.

**Keywords:** atypical hemolytic uremic syndrome (aHUS), complement inhibitor, eculizumab, systemic lupus erythematosus (SLE), thrombotic microangiopathy (TMA)

## Abstract

**Rationale:**

Systemic lupus erythematosus (SLE)-associated thrombotic microangiopathy with features of atypical hemolytic uremic syndrome (aHUS) is a rare but life-threatening complication. Early recognition is challenging because of its overlapping clinical manifestations with other thrombotic microangiopathies.

**Patient concerns:**

A 49-year-old man with SLE presented with anemia, thrombocytopenia, and multi-system involvement. Despite initial immunosuppressive therapy, his hematologic parameters progressively deteriorated, with hemoglobin decreasing to 89 g/L and the platelet count to 54 × 10^9^/L.

**Diagnoses:**

Laboratory investigations demonstrated elevated lactate dehydrogenase (501 U/L) and markedly increased soluble complement membrane attack complex (sC5b-9, >1790 ng/mL). ADAMTS13 activity was normal (75.41%), excluding thrombotic thrombocytopenic purpura. Based on the clinical and laboratory findings, the patient was diagnosed with SLE-associated aHUS.

**Interventions:**

Following the diagnosis, the patient received eculizumab, a monoclonal antibody targeting complement component C5.

**Outcomes:**

Within 6 days of eculizumab treatment, hemoglobin increased to 102 g/L, and the platelet count recovered to 108 × 10^9^/L, indicating a rapid hematologic response.

**Lessons:**

This case highlights that aHUS can occur as a severe complication of SLE, even in male patients. Complement-mediated thrombotic microangiopathy may coexist with and be triggered by immune hemolysis in SLE. Early recognition and prompt complement inhibition with eculizumab can produce an ultra-rapid hematologic response and may be critical for improving patient outcomes.

## 1. Introduction

Systemic lupus erythematosus (SLE) is an autoimmune disease that can exhibit multi-system damage with a variety of hematologic manifestations. Atypical hemolytic uremic syndrome (aHUS) is a subtype of thrombotic microangiopathy (TMA) with a rare onset, characterized by microangiopathic hemolytic anemia, thrombocytopenia, and acute kidney injury. The pathogenesis of aHUS is mainly due to inherited and acquired abnormalities in complement regulation. TMA and aHUS rarely coexist with SLE, which can lead to life-threatening complications if not recognized and treated in time.^[1]^ In this report, we describe a 49-year-old man with an initial diagnosis of SLE and exhibiting aHUS, who was rapidly controlled after the addition of the complement component 5 (C5) inhibitor eculizumab.

## 2. Case description

A 49-year-old male worker presented with a 2-month history of facial swelling with shortness of breath after activity and was hospitalized in the Second Affiliated Hospital of Fujian Medical University after unsatisfactory treatment in several hospitals. He underwent relevant examinations with the following results. Chest computed tomography: few possible chronic inflammatory lesions in the lower lobes of both lungs, pericardial effusion. Cardiac doppler ultrasound: moderate amount of pericardial effusion. Lower extremity venous vascular ultrasound: no significant thrombus. The laboratory indicators are shown in Tables 1 and 2.

**Table 1:** The Laboratory indicators for initial diagnosis.

Index	Result	Reference range	Unit
WBC	3.77	3.5–9.5	10^9^/L
NE%	79.3	40–75	%
NE#	2.99	1.8–6.3	10^9^/L
RBC	3.09	4.3–5.8	10^12^/L
HGB	97	130–175	g/L
PLT	71	125–350	10^9^/L
CRP	5.06	0–6	mg/L
ESR	122	≤15	mm/H
ALB	31.5	40.0–55.0	g/L
AST	81.3	15–40	U/L
GGT	100	10–60	U/L
CREA	0.6	0.6–1.1	mg/dL
NT-proBNP	734.7	0–300	pg/mL
D-D	1.9	0–0.55	µg/mL
C3	0.293	0.9–1.8	g/L
C4	0.042	0.1–0.4	g/L
Lupus anticoagulant	(−)	(−)	/
Coombs IgG	2+	(−)	/
urine occult blood	Weakly positive	(−)	/
Qualitative Urine Protein	2+	(−)	/
24-Hour Urine Protein Quantification	567	28–141	mg/24 h

ALB = albumin, AST = aspartate aminotransferase, C3 = complement component 3, C4 = complement component 4, CREA = creatinine, CRP = c,reactive protein, D-D = D-dimer, ESR = erythrocyte sedimentation rate, GGT = gamma,glutamyl transferase, HGB = hemoglobin, NE# = neutrophil count (absolute number), NE% = neutrophil percentage, NT-proBNP = N-terminal pro B-type natriuretic peptide, PLT = platelets, RBC = red blood cell, WBC = white blood cell.

**Table 2:** Autoantibody testing results.

Index	Result	Reference range	Testing method
Anti-PM/Scl antibody	0.1	<1	Chemiluminescence immunoassay
Anti-PCNA antibody	0.09	<1	Chemiluminescence immunoassay
Anti-M2 AMA	0.41	<1	Chemiluminescence immunoassay
Anti-U1-snRNP antibody	0.45	<1	Chemiluminescence immunoassay
Anti-Sm antibody	0.14	<1	Chemiluminescence immunoassay
Anti-native SS-A antibody	1.67	<1	Chemiluminescence immunoassay
Anti-recombinant Ro-52 antibody	1.49	<1	Chemiluminescence immunoassay
Anti-SS-B antibody	2.74	<1	Chemiluminescence immunoassay
Anti-Scl-70 antibody	0.27	<1	Chemiluminescence immunoassay
Anti-Jo-1 antibody	0.12	<1	Chemiluminescence immunoassay
Anti-CENP-B antibody	0.47	<1	Chemiluminescence immunoassay
Anti-dsDNA antibody	164.26	<100	Chemiluminescence immunoassay
Anti-nucleosome antibody	0.83	<1	Chemiluminescence immunoassay
Anti-histone antibody	1.01	<1	Chemiluminescence immunoassay
Anti-Rib-P antibody	2.05	<1	Chemiluminescence immunoassay
Anti-β2-glycoprotein I antibody	19.07	0–20	ELISA
Anti-cardiolipin antibody	(−)	(−)	ELISA
ANA (Speckled pattern)	>1: 1000	<1: 100	Indirect immunofluorescence assay
ANCA	(−)	(−)	Indirect immunofluorescence assay

AMA = anti-mitochondrial antibody, ANA = antinuclear antibody, ANCA = antineutrophil cytoplasmic antibody, CENP-B = centromere protein B, dsDNA = double stranded DNA, Jo-1 = histidyl-tRNA synthetase, PCNA = proliferating cell nuclear antigen, PM/Scl = polymyositis/scleroderma, Rib-P = ribosomal P protein, Ro-52 = Ro-52 (also known as TRIM21), Scl-70 = scleroderma-70, Sm = Smith, snRNP = small nuclear ribonucleoprotein, SS-A = Sjögren’s syndrome antigen A, SS-B = Sjögren’s syndrome antigen B, β2-glycoprotein I = beta-2 glycoprotein I.

The patient met the diagnostic criteria for SLE based on the following findings: positive ANA (speckled pattern, titer > 1:1000); positive anti-dsDNA antibody (164.26, reference range < 100); positive anti-Sm antibody, anti-SS-A antibody, anti-SS-B antibody, and anti-Rib-P antibody; hematologic involvement (anemia with hemoglobin 97 g/L, thrombocytopenia with platelets 71 × 10^9^/L); renal involvement (24-hour urine protein quantification 567 mg/24 h, weakly positive urine occult blood); pericardial effusion (moderate amount confirmed by cardiac ultrasound).

The patient was treated with methylprednisolone (80 mg/d intravenously for 7 days), hydroxychloroquine sulfate, and cyclophosphamide (0.2 g intravenously every other day). After treatment, his shortness of breath was significantly relieved, his mental state improved markedly, and he began to walk around the ward. However, despite these symptomatic improvements, a reevaluation after 7 days showed the following changes: complement levels increased slightly compared with the initial values (C3: 0.411 g/L, C4: 0.061 g/L); pericardial effusion decreased; hemoglobin and platelets continued to decline (hemoglobin: 89 g/L, platelets: 54 × 10^9^/L). Since the total dose of cyclophosphamide used was only 0.4 g, which was insufficient to cause myelosuppression, and the reticulocyte count (Ret) was 3.16% (indicating active erythropoiesis), other underlying causes were suspected.

A recheck revealed the following: D-dimer 3.61 μg/ml (reference range 0–0.55 μg/ml), lactate dehydrogenase (LDH) 501 U/L (reference range usually 109–245 U/L). These results raised suspicion of TMA. Subsequent testing showed a schistocyte percentage of approximately 5.3% (normal < 1%), further supporting the diagnosis of TMA. TMA includes several subtypes, such as TTP, Shiga toxin-associated hemolytic uremic syndrome (STEC-HUS), and aHUS. To differentiate between these subtypes: The patient had no gastrointestinal symptoms such as diarrhea, and his serum creatinine was normal (0.6 mg/dl), ruling out STEC-HUS; ADAMTS13 activity testing showed a value of 75.41% (normal > 60%), and ADAMTS13 activity inhibitory antibody was negative, ruling out TTP. Thus, the diagnosis of aHUS was confirmed.

Additional supporting evidence for aHUS included: MAHA (decreased hemoglobin, elevated reticulocytes, presence of schistocytes on blood smear, elevated LDH); thrombocytopenia; urinary abnormalities (weakly positive urine occult blood, 2 + qualitative urine protein); significantly elevated soluble complement membrane attack complex (C5b-9) > 1790 ng/mL (normal 75–219 ng/mL).

**Figure 1.**
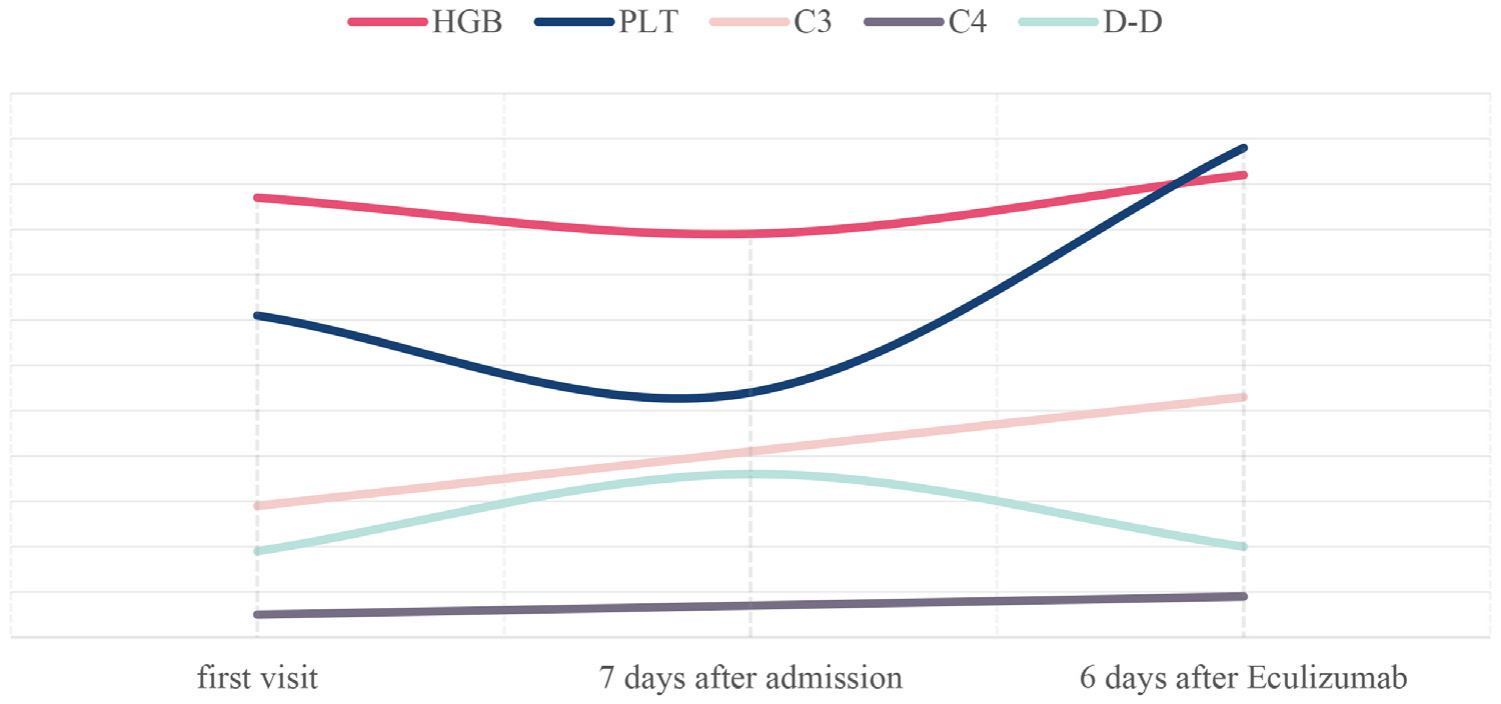
Dynamic changes of key laboratory indicators in the patient at different time points. The figure shows the detection results of 5 clinical laboratory indicators in the patient during the treatment process. HGB reflects the oxygen-carrying capacity of red blood cells; PLT is involved in blood coagulation and is related to bleeding/thrombosis risk; C3 and C4 are key members of the complement system, and their levels indicate the activation status of the immune system; D-D reflects the activation of the coagulation and fibrinolysis system. The 3 time points are defined as follows: “first visit” refers to the patient’s first medical consultation; “7 days after admission” refers to 7 days after the patient was hospitalized; “6 days after Eculizumab” refers to 6 days after the patient received Eculizumab treatment. All indicator values are presented in their respective routine clinical units (HGB: g/L; PLT: ×10^9^/L; C3, C4: mg/dL; D-D: μg/mL FEU). C3 = complement component 3, C4 = complement component 4, D-D = D-dimer, HGB = hemoglobin, PLT = platelet.

Eculizumab, a C5 complement inhibitor, was added to the treatment regimen. Within 6 days of administration, the patient’s condition was rapidly controlled: hemoglobin increased to 102 g/L, platelets increased to 108 × 10^9^/L; urinary protein remained 2+, and 24-hour urine protein quantification decreased to 485 mg/24h; renal function parameters (creatinine) remained stable; symptoms such as shortness of breath and edema improved significantly. The patient was discharged and scheduled for outpatient follow-up, with regular eculizumab treatment. Changes in key indicators during treatment are shown in Table 3 and Figure 1.

**Table 3:** Changes in some indicators during treatment.

TIME	HGB (g/L)	PLT (10^9^/L)	Ret (%)	C3 (g/L)	C4 (g/L)	D-D (ug/ml)	LDH (U/L)	Qualitative urine protein	Urine Protein Quantification	schistocyte percentage	ADAMTS13 activity	c5b-9 (ng/mL)
First visit	97	71	/	0.293	0.042	1.9	/	2+	567	/	/	/
7 d after admission (TMA suspected period)	89	54	3.16	0.411	0.061	3.61	501	/	/	5.3%	75.41%	>1790
6 d after Eculizumab	102	108	/	0.538	0.076	2.1	/	2+	485	/	/	/

ADAMTS13 = a disintegrin and metalloproteinase with a thrombospondin type 1 motif, member 13, C3 = complement component 3, C4 = complement component 4, C5b-9 = complement membrane attack complex, D-D = D-dimer, HGB = hemoglobin, LDH = lactate dehydrogenase, PLT = platelet count, Ret = reticulocyte count, TMA = thrombotic microangiopathy.

## 3. Discussion

1. Diagnostic challenge and differential diagnosis of TMA in SLE

The occurrence of TMA in SLE poses a significant diagnostic challenge, necessitating a swift and accurate differential diagnosis among TTP, STEC-HUS, and aHUS, as their management strategies differ considerably.^[2]^ In our patient, the absence of diarrhea argued against STEC-HUS. The preservation of normal renal function was atypical for classical aHUS but is documented in SLE-associated cases. The key differentiator was the normal ADAMTS13 activity (75.41%), which confidently excluded TTP and pointed towards aHUS. The diagnosis was further corroborated by the significantly elevated C5b-9, confirming uncontrolled complement activation. This case underscores the critical importance of a structured diagnostic workup, including ADAMTS13 testing and complement biomarkers, in SLE patients with new-onset MAHA and thrombocytopenia.

1. Pathophysiology: interaction between immune hemolysis and complement-mediated TMA

The pathophysiology of this case involves a mixed mechanism, which can be explained by the “two-hit model” of SLE-TMA and the interaction between immune hemolysis and complement activation: 1) First hit: immune complex-mediated endothelial injury: SLE is characterized by the formation of immune complexes, which deposit in blood vessels and activate the classical complement pathway, leading to endothelial damage.^[3]^ In this patient, the presence of hypocomplementemia (low C3 and C4) at the initial diagnosis indicated activation of the classical complement pathway by immune complexes. This endothelial injury creates a prothrombotic environment, laying the foundation for the development of TMA. Second hit: Complement-mediated TMA (aHUS): The “second hit” in this case was the overactivation of the alternative complement pathway, leading to the development of aHUS. The patient’s significantly elevated C5b-9 levels (>1790 ng/mL) indicated excessive activation of the terminal complement pathway, which is a key pathogenic feature of aHUS.^[4,5]^ Unlike primary aHUS (which is often caused by genetic mutations in complement regulatory proteins, such as complement factor H or I),^[6]^ the complement activation in this case was secondary to SLE activity, but it shared the same terminal pathway activation and endothelial damage as primary aHUS.

Coexistence of immune hemolysis and complement-dominant MAHA: The patient’s positive Coombs IgG test indicated immune hemolysis (mediated by autoantibodies against red blood cells), which is a common hematologic manifestation of SLE. However, the presence of schistocytes and elevated LDH suggested an additional mechanism of MAHA mediated by complement activation (aHUS). This coexistence is rare and highlights the complexity of SLE-associated TMA. SLE patients with TMA may have overlapping immune and complement-mediated mechanisms, and the distinction between primary aHUS, secondary SLE-TMA, and overlap syndromes requires careful analysis of laboratory markers (such as Coombs test, complement levels, and genetic testing).^[7]^

Regarding genetic testing for complement pathway variants: We considered performing genetic testing to identify potential inherited complement abnormalities that may have predisposed the patient to aHUS. However, due to the patient’s limited economic resources and the lack of immediate access to genetic testing services in our institution, this testing was not feasible. Future studies should include genetic testing in SLE patients with aHUS to determine the role of genetic factors in the pathogenesis of this complication.^[8,9]^

1. Therapeutic implications and deviation from standard guidelines

Current guidelines for severe SLE flares with TMA recommend pulse corticosteroids and plasma exchange (PLEX) as first-line therapy.^[10]^ Our decision to forgo pulse steroids and PLEX in favor of immediate eculizumab was individualized. This deviation was based on the patient’s rapid symptomatic improvement with moderate-dose steroids, the absence of immediately life-threatening neurological or renal crisis, and concerns about infection risk given significant leukopenia. The subsequent rapid hematologic response to eculizumab (within 6 days) validated the central role of complement in this case and is consistent with reports of its efficacy in SLE-TMA.^[11,12]^

However, it is crucial to emphasize that this approach may not be generalizable. In most cases of SLE-TMA, especially with severe organ involvement, high-dose corticosteroids and PLEX remain the standard initial therapy to broadly suppress inflammation and remove autoantibodies. Eculizumab should be considered an adjunctive or salvage therapy in refractory cases or when complement dysregulation is strongly implicated. The optimal duration of eculizumab in SLE-TMA is unknown. Genetic testing for complement variants could guide long-term management, but it was not feasible in this case due to resource constraints, representing a knowledge gap for future research.^[8,9]^

## 4. Conclusion

This case illustrates a severe presentation of SLE-associated TMA with aHUS features, where targeted complement inhibition with eculizumab rapidly reversed life-threatening hematologic manifestations. It highlights the diagnostic challenges and the potential synergy between immune hemolysis and complement activation in SLE. Our experience suggests that eculizumab can be a highly effective intervention in select cases. However, significant knowledge gaps remain regarding the optimal timing for initiating complement blockade, the criteria for its discontinuation, and the role of underlying genetic susceptibility. Future research focusing on complement genetics and biomarkers is needed to personalize therapy and improve outcomes for patients with this complex and overlapping syndrome.

## Author contributions

**Supervision:** Jinjian Guo.

**Validation:** Jinjian Guo.

**Writing – original draft:** Yuting Lin.
